# Scapular winging beyond shoulder pathology: a two-case series highlighting neurological red flags for surgeons

**DOI:** 10.1093/jscr/rjag484

**Published:** 2026-06-22

**Authors:** Sebastian Radmer, Julian Ramin Andresen

**Affiliations:** Centre for Orthopaedics, Bozener Str. 17, 10825 Berlin, Germany; University Clinic for Orthopaedics and Trauma Surgery, Medical University of Vienna, Währinger Gürtel 18-20, 1090 Vienna, Austria

**Keywords:** scapular winging, syringomyelia, neuralgic amyotrophy, Parsonage-Turner syndrome, long thoracic nerve, differential diagnosis

## Abstract

Scapular winging is an uncommon but clinically important sign that is often initially attributed to local shoulder pathology or isolated peripheral nerve injury. We report two patients with exertional scapular winging caused by different neurological disorders that are relevant to orthopaedic and surgical practice. The first patient presented with shoulder pain, weakness, sensory disturbance, and a reduced biceps reflex. Electrophysiology demonstrated a C6 radicular syndrome, and cervical magnetic resonance imaging revealed syringomyelia at the C6 level. The second patient developed acute nocturnal shoulder pain followed by persistent weakness and scapular winging. Electrophysiology and nerve ultrasound showed a long thoracic nerve lesion consistent with neuralgic amyotrophy. Both patients were treated conservatively. Pain improved, but scapular winging persisted. These cases show that scapular winging is a clinical sign with a broad differential diagnosis and that structured neurological assessment is essential to avoid misdiagnosis and inappropriate local treatment.

## Introduction

Scapular winging is an uncommon but clinically relevant sign that may cause pain, weakness, impaired shoulder function, and visible deformity. It usually reflects dysfunction of the scapulothoracic stabilizers, most commonly the serratus anterior, trapezius, or rhomboid muscles [1].

In routine clinical practice, scapular winging is often initially attributed to peripheral nerve injury, particularly involving the long thoracic nerve, or to local shoulder pathology [2]. However, scapular winging has a broader etiological spectrum and may also result from more complex neurological disease [1, 2].

This distinction is especially important in orthopaedic and surgical settings, where a visible scapular deformity may prompt local investigations or interventions while the actual cause lies within the cervical spine, brachial plexus, or peripheral nervous system. We report two patients with clinically manifest scapular winging caused by distinct neurological disorders. The first patient had cervical syringomyelia presenting with a radicular pattern, whereas the second had neuralgic amyotrophy involving the long thoracic nerve. The purpose of this two-case series is to highlight neurological red flags that should prevent misdiagnosis and inappropriate local treatment.

## Case series

### Case 1

**Figure 1:**
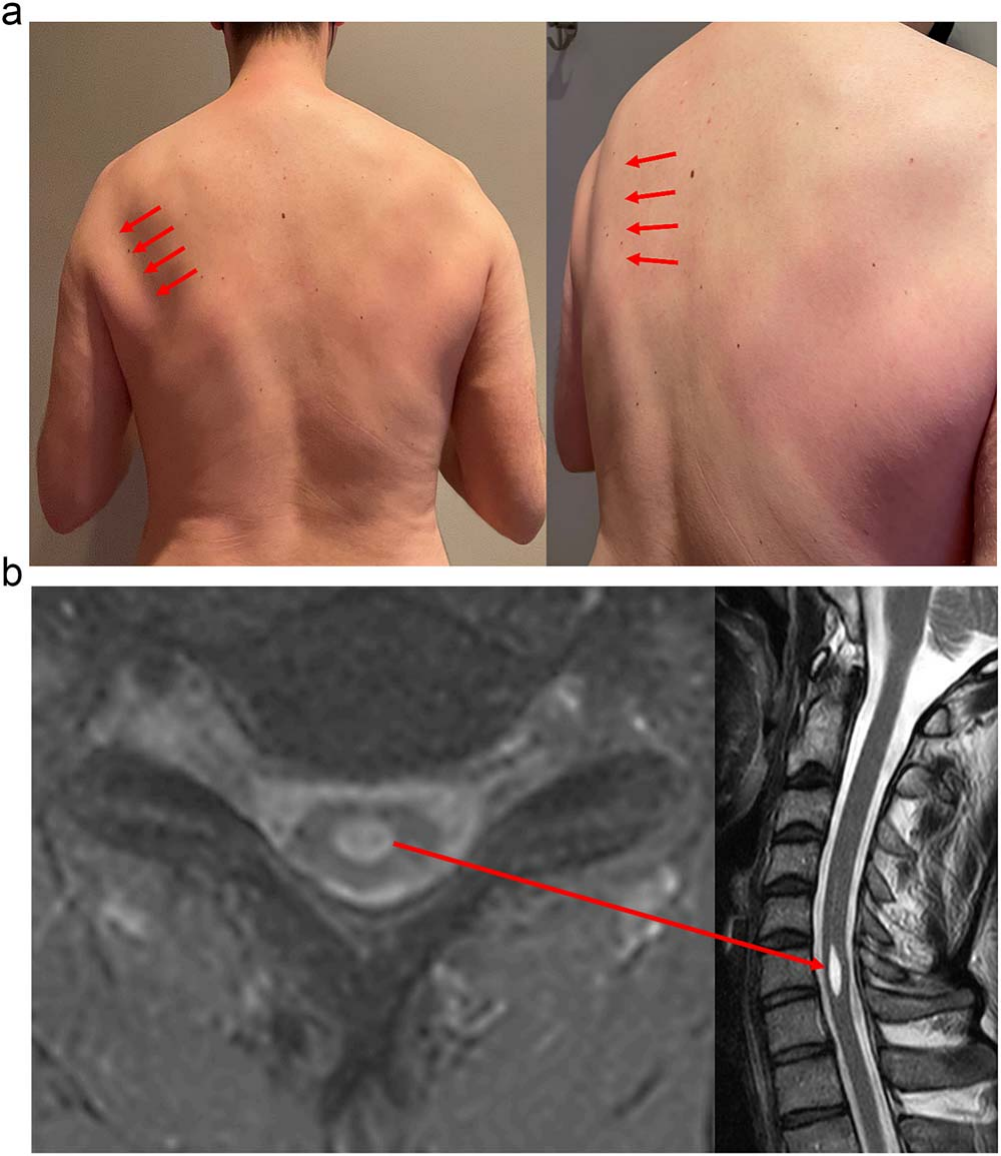
(a) Clinical image of scapula alata with slight winging of the left scapula (marked by arrows). (b) MRI of the cervical spine showing cervical syringomyelia at the level of the sixth cervical vertebra with a craniocaudal extension of ~20 mm. The syringomyelia appears hyperintense on the axial mFFE image (left) and sagittal T2-weighted image (right); the lesion is marked with an arrow.

A 36-year-old man presented with progressive shoulder pain, exertional scapular winging (Fig. 1a), weakness, and sensory disturbance of the left arm. Clinical examination showed mild atrophy of the latissimus dorsi muscle, accentuation of scapular winging during a wall push-up test, a reduced biceps tendon reflex, and dysesthesia in the C6 dermatome. Laboratory testing showed no evidence of inflammation, and Lyme disease was excluded. Plain radiographs of the cervical spine and shoulder showed no relevant abnormality.

Electrophysiological assessment demonstrated a C6 radicular syndrome with paresis of the latissimus dorsi, biceps brachii, and rhomboid muscles. Because of the associated neurological findings, contrast-enhanced magnetic resonance imaging (MRI) of the cervical spine was performed. This revealed cervical syringomyelia at the C6 level with a longitudinal extension of 2 cm (Fig. 1b). Disc herniation, relevant spinal canal stenosis, and neoplastic disease were excluded.

The patient was treated conservatively with physiotherapy focusing on strengthening, stretching, and functional stabilization, together with non-steroidal anti-inflammatory drugs as required. During follow-up, pain improved, but scapular winging persisted.

### Case 2

**Figure 2:**
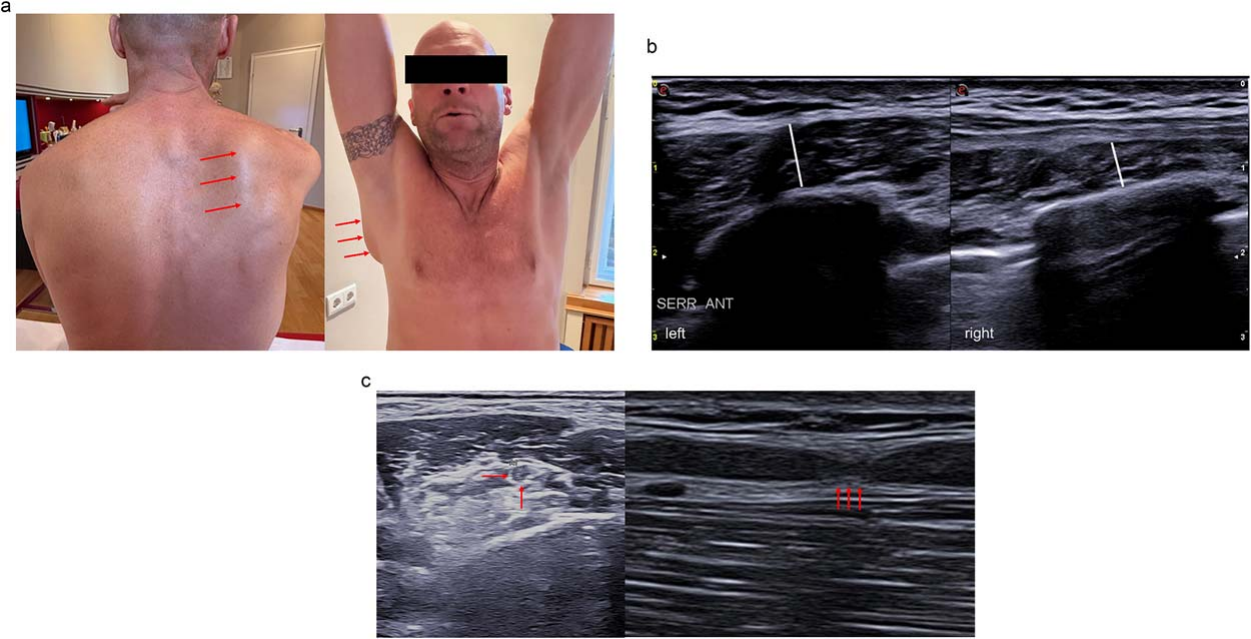
(a) Clinical image of right scapula alata with elevation of the medial scapular border (marked with arrows). (b) Sonography showing slight atrophy of the right serratus anterior muscle. (c) High-resolution ultrasound showing perifocal thickening in the axial view (arrows) and an hourglass-shaped nerve constriction (arrows) in the longitudinal view of the long thoracic nerve.

A 50-year-old man presented with acute severe nocturnal right shoulder pain that gradually subsided over the following weeks. He subsequently developed persistent weakness of the right arm, sensory disturbance over the lateral upper arm, and increasing exertional scapular winging (Fig. 2a). Clinical examination showed mild atrophy of the serratus anterior muscle (Fig. 2b), and the wall push-up test confirmed scapular winging.

Electromyography and nerve conduction studies demonstrated a severe incomplete axonal lesion of the long thoracic nerve with corresponding weakness of the serratus anterior muscle. Brachial plexus and cervical root lesions were excluded. Nerve ultrasound showed mild focal enlargement of the long thoracic nerve (Fig. 2c), consistent with neuralgic amyotrophy. MRI of the cervical spine showed no disc pathology or myelopathy. MRI of the shoulder demonstrated mild volume loss of the serratus anterior muscle without marked fatty degeneration.

Laboratory testing showed no evidence of inflammatory disease. Infectious causes, including Lyme disease, HIV, herpes simplex virus, varicella-zoster virus, Epstein–Barr virus, hepatitis viruses, and COVID-19, were excluded. Treatment consisted of physiotherapy focusing on strengthening of the serratus anterior and scapular stabilizers, together with stretching and relaxation exercises. In the acute phase, in addition to adequate pain therapy (oxycodone/naloxone 10/5 mg for 7 days), oral corticosteroid therapy with prednisolone (60 mg/daily for 1 week, then 50, 40, 30, 20, 10, and 5 mg for 6 days) was also administered. Pain improved during follow-up, but scapular winging remained.

## Discussion

This two-case series illustrates that scapular winging is not a diagnosis in itself but a clinical sign with a broad differential diagnosis. For orthopaedic surgeons and other clinicians dealing with shoulder complaints, this distinction is critical because a similar external appearance may arise from fundamentally different neurological mechanisms.

In the first case, the underlying cause was cervical syringomyelia. Syringomyelia is a rare disorder characterized by fluid-filled cavities within the spinal cord that may produce variable neurological deficits depending on their location and extent. In this patient, the key diagnostic clues were the combination of scapular winging with dermatomal sensory disturbance, reflex asymmetry, and electrophysiological evidence of radicular involvement. These findings prompted cervical imaging and led to the correct diagnosis. The case demonstrates that scapular winging should not automatically be interpreted as an isolated peripheral nerve disorder or a local shoulder problem when additional neurological red flags are present.

In the second case, the clinical and instrumental findings were consistent with neuralgic amyotrophy, also known as Parsonage-Turner syndrome [3]. The disorder is generally regarded as an immune-mediated inflammatory neuropathy, with infections, surgery, and vaccination among the reported triggers [4]. Because nerves outside the brachial plexus may also be affected, the broader term neuralgic amyotrophy is increasingly preferred [4, 5]. The typical clinical course consists of acute severe pain followed by weakness and muscle wasting. When the long thoracic nerve is involved, serratus anterior weakness results in scapular winging. Imaging and high-resolution nerve ultrasounds are increasingly important in the diagnostic work-up [6, 7].

From a practical perspective, both cases support a stepwise diagnostic approach to scapular winging. The history may already suggest the likely mechanism: acute nocturnal pain followed by weakness strongly supports neuralgic amyotrophy, whereas progressive symptoms combined with reflex changes or dermatomal sensory disturbance should raise suspicion of cervical root or spinal cord pathology. Clinical examination should define the pattern of muscle weakness and identify associated neurological deficits. Electrophysiological studies help differentiate radiculopathy, plexopathy, and peripheral mononeuropathy. Targeted imaging of the cervical spine, shoulder region, and, where appropriate, the affected peripheral nerve may then reveal structural abnormalities relevant to diagnosis and management [6, 7].

For surgeons and orthopaedic specialists, this distinction has direct therapeutic implications. Visible scapular deformity may otherwise be misinterpreted as a local shoulder disorder, potentially leading to inappropriate operative or interventional treatment. Conversely, correct diagnosis may prevent unnecessary local procedures and redirect management toward functional rehabilitation and neurological evaluation.

Treatment depends on the underlying etiology. In both patients, management was conservative. In neuralgic amyotrophy, oral corticosteroids are commonly used in the early phase, although evidence remains limited and motor recovery is often slow and incomplete [8]. In selected patients with persistent symptoms and structural nerve constriction, surgical decompression or microneurolysis may be considered [9]. In our second case, this option was discussed but declined by the patient. By contrast, treatment options in syringomyelia depend largely on the underlying cause and the presence of neurological progression. Taken together, these cases emphasize that the central clinical challenge is not scapular winging itself, but recognition of the underlying neurological disorder.

## Conclusion

Scapular winging should be regarded as a clinical sign rather than a diagnosis and warrants careful evaluation of its underlying cause. In addition to long thoracic nerve dysfunction, central neurological disorders such as cervical syringomyelia must also be considered. When reflex abnormalities, dermatomal sensory deficits, or electrophysiological evidence of radiculopathy are present, extended neurological assessment is essential to avoid misdiagnosis and inappropriate local treatment in orthopaedic and surgical practice.

## Data Availability

Data sharing is not applicable to this article as no new datasets were generated or analysed.
